# Patient navigation programmes for symptomatic lung cancer patients: a scoping review

**DOI:** 10.1186/s13690-025-01786-y

**Published:** 2025-11-26

**Authors:** Elysse Bautista-Gonzalez, Heber Tomas Reyes-Garcia, Regina Barragan-Carrillo, Bertha Alejandra Martinez-Cannon, Andrés Quintero-Leyra, Hynek Pikhart, Anne Peasey, Cecilia Vindrola-Padros

**Affiliations:** 1 Research Department of Epidemiology and Public Health, Institute of Epidemiology and Health Care, University College London, London, UK; 2 Centro Médico Nacional 20 de Noviembre del ISSSTE, Ciudad de México, México; 3 Medical Oncology Department, National Cancer Institute (INCan), Ciudad de México, México; 4 Hemato-Oncology Department, Instituto Nacional de Ciencias Médicas y Nutrición Salvador Zubirán, Ciudad de México, México; 5 Public Health Department, Faculty of Medicine, National Autonomous University of Mexico, Ciudad de México, México; 6 Rapid Research Evaluation and Appraisal Lab, University College London, London, UK; 7 Research Department of Targeted Intervention, Division of Surgery and Interventional Science, Central London Patient Safety Research Collaboration (PSRC), University College London, London, UK

**Keywords:** Health services research, Patient navigation, Lung cancer, Scoping review

## Abstract

**Introduction:**

This scoping review investigates the landscape of patient navigation programs (PNP) specifically for lung cancer, addressing a gap in existing literature that has predominantly focused on other cancers or chronic diseases and screening.

**Methods:**

Employing Arksey and O’Malley’s framework and PRISMA-ScR guidelines, we aimed to characterize the features of lung cancer PNP, understand their outcomes, and analyse the applied methodologies for research. The search was conducted in January 2025.

**Results:**

The search led to the inclusion of 16 studies, predominantly from high-income countries, revealing a variety of implementation strategies and navigator types. Findings indicate a significant emphasis on administrative outcomes, clinical and patient-reported outcomes with heterogeneity in its use. Emotional support, logistical assistance, and healthcare system navigation emerged as key themes in service delivery, however PNP focus is inconsistent. The analysis highlighted the need for standardized methodologies and outcome measures, as well as greater inclusivity in research populations. Importantly, the review identified a trend of excluding marginalized demographics, raising concerns about the generalizability and external validity of findings.

**Conclusion:**

Our study underscores the necessity for future research to harmonize PNP methodologies, enhance transparency in collaborative efforts, and focus on addressing health inequalities to optimize the effectiveness of lung cancer navigation initiatives.

**Supplementary Information:**

The online version contains supplementary material available at 10.1186/s13690-025-01786-y.

**Table:** 

Text box 1. Contributions to the literature
• Patient navigation for lung cancer is more predominantly used for screening and from diagnosis to treatment.
• Many programmes frequently use quasi-experimental design and are paired with other cancer types, limiting the analysis of the outcomes and effectiveness.
• Patient navigation programs for symptomatic lung cancer patients vary substantially in their implementation.
• Outcomes usually assessed include patient reported , clinical and administrative outcomes.
• There isn’t sufficient evidence to support there is reduction in delays due to patient navigation programme implementation.
• Future research should focus on strengthening research practices, harmonizing patient navigation implementation.

## Background

The concept of “patient navigation” was initially developed in the United States of America (USA) as a strategy to address the disparities in cancer outcomes between low-income, minority, and immigrant populations [1, 2]. In 1989, the “Report to the Nation on Cancer in the Poor” found that people that have been economically marginalized faced substantial barriers to obtaining cancer care, leading to great hardship. In response to such efforts, Harold Freeman pioneered the first patient navigation program among breast cancer patients in Harlem, New York, in 1990 [1].

Patient navigation generally targets individuals and communities most at risk for experiencing barriers, with the goal “to facilitate timely access to quality cancer care that meets cultural needs and standards of care for all patients” [3]. The navigation process can begin at any point in time. There are three main actors in the navigation process: the navigator, the user (patient or family member), and the medical team embedded within the health system. Navigators become patient advocates that help patients overcome barriers in the health system through collaborative efforts with their caregivers or family members [4, 5].

Patient navigation programmes (PNP) have four basic elements: case identification, detection of structural and individual barriers, development of a personalized plan to address those barriers [4, 6], and systematic follow-up to track progress across the health continuum [3, 7]. Unlike case management, which organizes patient care activities and services with different providers and seeks quality of service, optimal utilization, and lower costs, PNP aims to reduce health inequalities by considering the patient’s context and perspective [4]. Although elements of patient navigation frequently overlap with managed care, case management, advocacy, and social work, navigation is distinguished from these services by its focus on identifying and addressing logistical, psycho-social, and personal barriers to care [1, 4, 6, 8].

Many researchers have studied patient navigation for surgery [9], chronic diseases [10, 11], cancer [12], or even paediatric illness [13]. For cancer particularly, studies have found substantial increases in timely cancer screening [14–17]. Other studies infer an increase in screening uptake or an increase in early-stage diagnosis [14, 18] and opportune treatment [18, 19]. Additionally, PNP services seem to play a crucial role in enhancing the patient’s satisfaction and clinical health outcomes [20]. In consequence, patient navigation programmes were suggested to be implemented in Latin America to improve cancer control [21]. Nonetheless, for lung cancer particularly, there is not a vast amount of evidence of its effect in early diagnosis isolated from other types of cancer [22]. Only one systematic review was found aiming to identify patient navigation’s effect for lung cancer screening and not early diagnosis and treatment [22]. Thus, more research is needed to explore the existence of patient navigation for symptomatic patients, its main characteristics and reported effects to assess the uptake of this recommendation. To our knowledge, no scoping review has ever been conducted specifically for navigation of lung cancer patients beyond screening for symptomatic patients.

## Methods

### Search

The scoping review was based on the Arksey and O’Malley’s methodology framework [23] and the Preferred Reporting Items for Systematic Review and Meta-Analyses extension for Scoping Reviews (PRISMA- ScR) [24].

To identify peer-reviewed literature, we searched PubMed, Medline, Global Index Medicus, and Web of Science on January 24, 2025. The main intervention sought was patient navigation for patients diagnosed with lung cancer (from symptom onset to treatment). The search strategy used for each database is detailed in Table 1.

**Table 1:** Search strategy used in each database for the scoping review “Patient navigation programmes for symptomatic lung cancer patients” (January 2025)

Source	Search strategy
PubMed	(((“lung cancer*“[Title]) OR (“lung tumo*“[Title])) OR (“lung neoplasm“[Title])) AND (“patient navigation program*“[Title] OR “Patient navigation“[Title]))
MEDLINE	((“lung cancer*” or “lung tumo*” or “lung neoplasm”) and (“patient navigation program*” or “Patient navigation”)).ti.
Global index Medicus	(ti: (“lung cancer”)) OR (ti: (“lung neoplasm”)) OR (ti: (“lung tumour”)) OR (ti: (“lung tumor”)) AND (ti: (“Patient navigation”))
Web of Science	(((TS=(“lung cancer”)) OR TS=(“lung neoplasm”)) OR TS=(“cancer de pulmon”) OR TS=(“lung tumour”)) AND TS=(“Patient navigation”)

### Research questions

1. What are the main characteristics of PNP navigating patients?
  Disease navigated (lung cancer or other diseases)
  Intervension(s) (Patient navigation or other implemental, clinical or administrative activities)
  Applied research methodology (Main outcomes),
  Reported impact of outcomes
2. What are the main recommendations for research on patient navigation for lung cancer?

### Study inclusion and exclusion criteria

PNP focusing on screening were not eligible for inclusion. Studies for which the population included paediatric patients were excluded. All types of study designs were eligible. Control groups were not required to be included in the review, but if found, they were reported. Results were not mandatory for inclusion. However, systematic reviews and meta-analyses were excluded from the searches. Abstracts, conference papers, and trial registries were also excluded from the search. Furthermore, articles not in English and Spanish were excluded from the search. All authors involved in data collection and analysis were fluent in English and Spanish. A range of text words and terms related to “patient navigation” and “cancer” were used in these searches.

References were imported to Rayyan [25] and duplicates were deleted. Four additional researchers were supervised and trained by first author (EBG) to decide whether the studies met the inclusion/exclusion criteria. Studies that met the criteria were selected for inclusion in the review, while those that did not were rejected.

## Data extraction strategy

A data extraction spreadsheet was designed and piloted by *EBG* for the evidence synthesis process. Data was extracted by *EBG*,* AQL*,* RBC*,* BAMC*, and any disagreements were resolved by discussion or by involving a third reviewer until consensus was reached. Through scoping review methodology [26], the extracted data included: the characteristics of the navigated sample, the disease focus, the applied methodology and the description of the navigation intervention; authors screened the retrieved references for eligibility in each search engine.

### Data synthesis and presentation

Quantification of qualitative data was employed to capture the frequency of the emergence of PNP per year, across the globe, by type of cancer, population studied, and navigator type. Additionally, data comparison was conducted to capture differences in the intervention, activities conducted, and type of navigator employed by PNP. Studies were classified either as experimental (usually randomised controlled trials or RCT), observational, or quasi-experimental [27, 28]. Similarly, all PNP found were compared regarding the type of outcome measurements employed in each study (i.e., clinical, patient-reported outcomes, administrative). This included the description of survey tools employed, if any. Furthermore, a narrative synthesis of the results was used to discuss and compare the effect on clinical, administrative, and non-clinical outcomes. All findings were initially reviewed by *EBG* and subsequently re-evaluated by a different set of co-authors to ensure accuracy and consistency. Results were discussed between co/authors to identify discussion points, knowledge gaps and develop actionable recommendations for lung cancer patient navigation implementation and research.

## Results

**Fig. 1:**
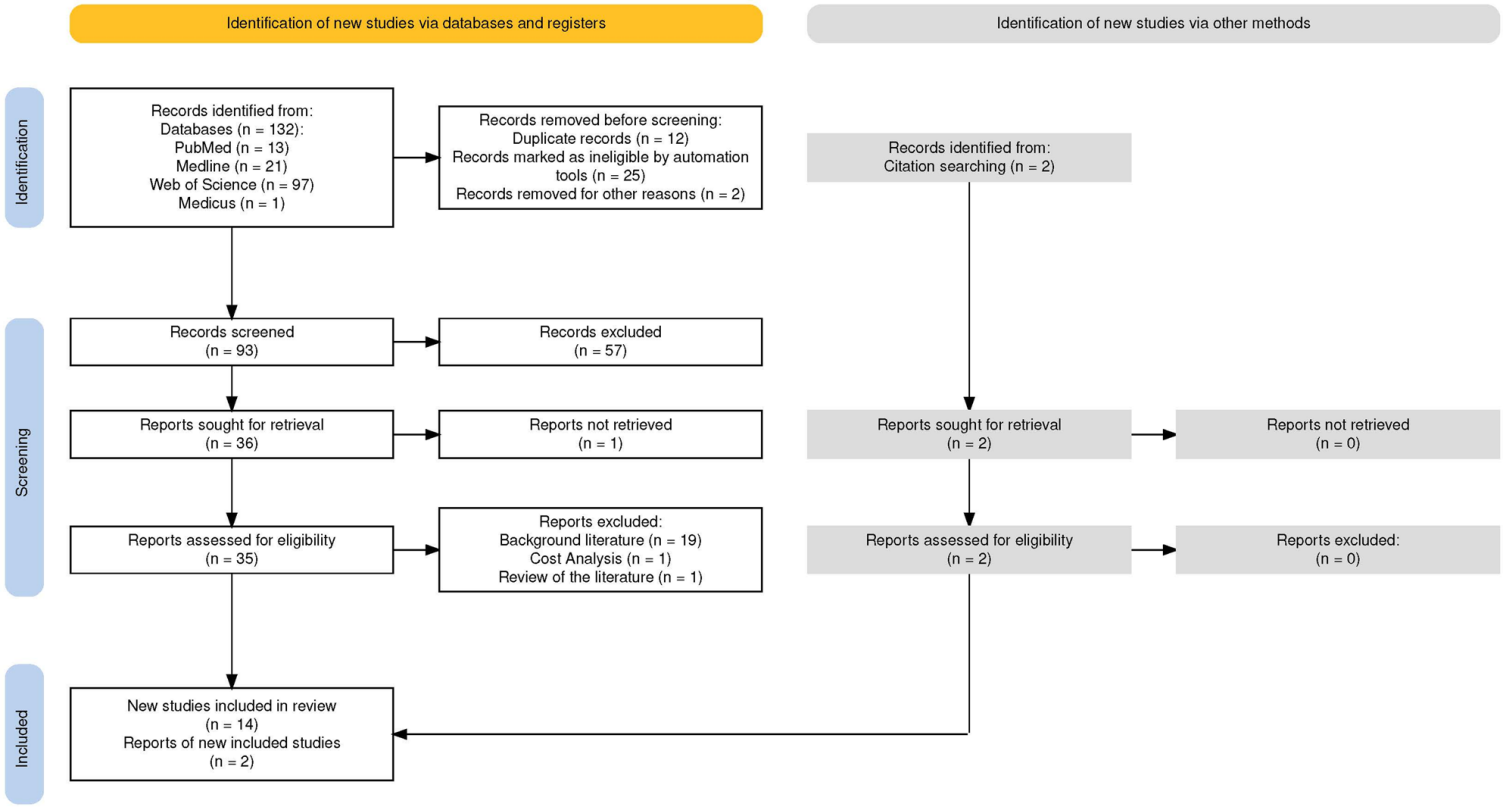
PRISMA results for the scoping review “Patient navigation programmes for symptomatic lung cancer patients” conducted in January 2025

Initially, 132 records were identified from databases such as PubMed, Web of Science, Medicus, and Medline, along with two additional records from citation searching. After removing duplicates, literature in languages other than English and Spanish; and systematic reviews or meta-analysis, 93 records were marked as eligible for screening, and only 36 of those were sought for retrieval, with one article not retrieved. Subsequently, 19 articles were excluded due to being background literature, cost analysis, and reviews of the literature. Ultimately, 16 studies were included in the review. See Fig. 1.

### General overview

Table 2 shows a general overview of the articles eligible for the review (*N* = 16). Our results show articles on patient navigation for lung cancer (from symptom to treatment) are commonly conducted in developed countries in the Northern Hemisphere: nine articles are from the USA, two from Canada and two from Denmark, while and Germany, the Netherlands, and Hungary each had one publication on PNP for lung cancer. Universities, foundations, and local hospitals frequently collaborated in crafting the programmes. The articles do not explicitly outline responsibilities and role distribution.

**Table 2:** Cancer types, publication year and country of origin extraction for the scoping review “Patient navigation programmes for symptomatic lung cancer patients” (*N*=16)

First Author	Year	Country	Patients studied	Study Design
Alsamarai S. et al.	2013	USA	Lung cancer	Quasi-experimental - retrospective pre-post design with external control cohort
Battaglia T.A. et al.	2022	USA	Lung & Breast	RCT
Berezowska A. et al.	2021	Netherlands	Lung, Renal, Ovarian, Vulvar, Endometrial Cancer & Melanoma	RCT
Charlot M. et al.	2022	USA	Lung Cancer	Quasi-experimental study
Chu J.N. et al.	2021	USA	Lung cancer, Colon Cancer, Liver cancer	Quasi-experimental - Feasibility study
Cykert S. et al.	2020	USA	Lung & Breast	Quasi-experimental
Fleisher L. et al.	2012	USA	Lung, Breast, Prostate, Colon & Cervical Cancer	Quasi-experimental study
Godde K. et al.	2023	Germany	Lung cancer & Stroke (Acute Brain Infarction)	RCT- Feasibility (Randomized Controlled Hybrid Type II Implementation-Effectiveness Study)
Griesemer I. et al.	2023	USA	Lung & Breast	Observational - Qualitative
Hunnibel L.S. et al.	2011	USA	Lung cancer	Quasi-experimental
Kjaer T.K. et al.	2017	Denmark	Lung cancer	Quasi-experimental
Langballe R. et al.	2022	Denmark	Lung cancer	Quasi-experimental
Lorhan S. et al.	2014	Canada	Lung cancer	Observational - concurrent mixed methods design
Pitter J.G. et al.	2022	Hungary	Lung cancer	Quasi-experimental study - retrospective cohort study with historical control
Wagner E.H. et al.	2014	USA	Lung, Breast & Colon	RCT
Zibrik K. et al.	2013	Canada	Lung cancer	Quasi-Experimental study - prospective pre-post design

#### Type of navigator for symptomatic lung cancer patients

Navigation services are provided by nurse navigators, specialised navigators (i.e., legal navigators), clinicians or lay navigators. In most cases, navigators were either health professionals with experience in the medical field or had some clinical training. The patient navigation programs were almost half the time studied together with other cancer types or chronic diseases. The most common pairing was with breast cancer patients.

#### PNP activities for symptomatic lung cancer patients

The intervention proposed by each article is described in Table 2. This table shows that PNP varied substantially in their implementation, frequency, activities, profile of the navigators, and location. While some involved only brief, remote communication between the patient and navigator, consisting mainly of information, advice, and encouragement, others involved much more extensive interaction and multiple in-person meetings. In some cases, the navigator may accompany the patient on visits or interface directly with healthcare providers, insurers, and others on the patient’s behalf. Training was also, in some cases, provided as part of the intervention. Additional details extracted on each article can be found in the Supplementary Table.

#### PNP study design for symptomatic lung cancer patients

Studies were not always RCT. In some cases, some studies were developed as feasibility studies [29–31]. These were rather more frequently using quasi-experimental design (pre-post with non-equivalent control), or observational. Feasibility studies reported challenges during the design or execution of the study itself [29–31]. Kjær et al. [31] designed a study aiming to improve receipt of stage-appropriate first-line treatment through a volunteer patient navigation intervention. However, the study failed to meet enrolment goals during the pilot due to multiple barriers, including patient reluctance (often tied to limited perceived benefit), poor timing of recruitment amid the emotional and logistical burden of initial diagnosis, and insufficient institutional resources to support recruitment processes [31]. Similarly, Godde et al. [30] described a feasibility study (CoreNAVI) which aimed to navigate patients with lung cancer and stroke. The authors anticipated several operational challenges, notably the fragmented German healthcare system, the complexity of implementing a multifaceted navigation model, and the absence of blinding due to the intervention design [30]. Recognizing these barriers, the study emphasized the importance of detailed process evaluation to understand patient acceptance, demand for services, and the practicality of integrating navigation into routine care [30]. Lastly, Chu et al. [29] also designed a feasibility study, and brought together a small sample of cancer patients. 

### Outcome priorities of PNP for symptomatic lung cancer patients

Outcomes such as patient satisfaction, quality of life, self-efficacy, self-activation, distress, trust in medical care, health costs, and supportive care use were of frequent interest to researchers [30, 32–34]. A diverse set of survey tools aimed at comprehensively assessing various aspects of patient experience and outcomes. The instruments employed included the Cancer Needs Distress Inventory (CaNDI) [34], which gauges distress levels and requirements specific to cancer patients, alongside general patient satisfaction measures. Additionally, the review incorporated the use of a survey and the distress thermometer to evaluate broader socio-legal aspects and distress levels. Furthermore, the European Organisation for Research and Treatment of Cancer Quality of Life Questionnaire (EORTC QLQ-C30) [35–37] was utilised in conjunction with the distress thermometer, the Symptom Management Self-Efficacy Scale tailored for breast cancer patients, and the Patient Satisfaction (Cancer Care Scale) to comprehensively assess quality of life, self-efficacy, and patient satisfaction. Finally, the medical consumption questionnaire and the Vulnerability Screening Instrument were employed to capture aspects related to healthcare resource utilisation and vulnerability screening, respectively [32]. This array of survey tools facilitated a comprehensive evaluation of PNP across multiple dimensions, providing valuable insights into their effectiveness and impact on diverse patient outcomes. Table 2 shows the type of outcomes assessed, including the use of patient reported outcomes, clinical and/or administrative outcomes. The most relevant findings are summarised below.

#### Patient-reported outcomes

Wagner et al. [38] tested a nurse navigator intervention in a cluster-randomized controlled trial with 251 patients newly diagnosed with breast, colorectal, or lung cancer, compared to enhanced usual care over 4 months. While the intervention did not significantly impact quality of life in the Functional Assessment of Cancer Therapy General scale (FACT-G scale), patients reported significantly better care experiences, including improved psychosocial support, care coordination, and information provision Patient Assessment of Chronic Illness Care (PACIC) and Picker subscales. There were no significant differences in time to oncology visits or treatment initiation, though control patients had surgery 6 days earlier on average.

Berezoska et al. [33], through an RCT demonstrated no significant effects on health-related quality of life, distress, or self-care knowledge compared to the control group. Nonetheless, in the patient navigation group they found lower consumption of supportive cancer care services, higher levels of self-efficacy, especially in fitness, employment, and sexuality, and higher levels of satisfaction with answers, advice, and empathy received from healthcare professionals [33].

A mixed-methods study by Lorhan et al. [39] focused on lung cancer and in understanding the barriers themselves in addition to measuring patient satisfaction with the PNP. There were only 29 participants in the evaluation of patient satisfaction using a Likert scale. However, through the qualitative arm, this research evidence increased continuity from primary to tertiary care and higher patient satisfaction [39].

Furthermore, a multicentre trial of a nurse navigator program was published by Langballe et al. [32]. Main outcomes of study were treatment adherence, self-activation, time to treatment, survival, quality of life using the European Organisation for Research and Treatment of Cancer Quality of Life Questionnaire for lung cancer, (EORTC QLQ-LC13) and EQ-5D-5 L, process evaluation, psychosocial outcomes, symptom burden, data completeness/follow-up, physical function tests, and health costs. Although no results are yet published, they expressed concerns over the potential for increased anxiety due to frequent collection of patient-reported outcomes.

#### Health inequalities

Only a few patient navigation studies focused on reducing health inequalities [40–42]. For instance, by employing qualitative methodologies, Griesemer et al. examined the ACCURE’s nurse navigation programme. It particularly focused on the reduction of differences in treatment completion rates between Black and White counterparts [43]; finding positive results. In other studies, the social determinants of health are somewhat considered by adjusting for race, age, insurance status, and other social socio-demographic characteristics. However, it is not the focus of their design and implementation.

#### Timely lung cancer care

Charlot et al. showed positive results, evidenced a reduction in time to surgery (from 34 days in retrospective control, 33 days in concurrent control, to 23 days in intervention) [42]. Additionally, the study found the proportion of patients being treated within 56 days increased (73% retrospective control, 72% concurrent control to 86% intervention), higher likelihood of patients being treated before 56 days (1.14 odds ratio in all intervention vs. all retrospective controls and 1.16 odds ratio in all intervention vs. all concurrent controls, *p* < 0.01) and higher likelihood of patients being treated before 42 days (1.23 odds ratio all intervention vs. all retrospective controls and 1.20 odds ratio in all intervention vs. all concurrent, *p* < 0.01) [42]. However, this study only included patients in stages I and II of the disease and with Non-Small Cell Lung Cancer diagnosis, specifically [42].

Hunnibell et al. [44] conducted a quasi-experimental study assessing the impact of an advanced practice nurse led navigation program on lung cancer diagnostic timelines in a vulnerable USA population from 2007 to 2010. The time from suspicion to treatment improved by 65 days, with smaller reductions in computed tomography (CT) and Positron emission tomography (PET) turnaround times. Although causality was not formally tested, the findings highlight the potential of advanced practice nurse led navigation in streamlining lung cancer diagnostics.

Similarly, Alsamarai et al. [45] conducted a retrospective quasi-experimental study at the Veterans Affairs Connecticut Healthcare System, comparing patients with non-small cell lung cancer managed through a cancer-specific care coordination program to those receiving standard care. The program was associated with significant improvements in timeliness of care, including a 25-day reduction from initial abnormal imaging to treatment initiation (*p* = 0.015), and a 23-day reduction from imaging to diagnosis (*p* = 0.016). Time from imaging to diagnosis also improved notably for patients with stage I cancers (131 to 87 days, *p* = 0.013) and for incidentally detected cancers (114 to 86 days, *p* = 0.031). Additionally, the program increased the proportion of patients diagnosed at early stages (I/II) from 32% to 48% (*p* = 0.006).

Zibrik et al. evaluated quality assurance and timeline endpoints, pre- and post-nurse navigation program implementation for triaging patients with thoracic malignancies [46]. Integrating a nurse navigator into the triage process increased the proportion of patients receiving systemic therapy and reduced the time to start systemic therapy by a median of 10 days. Rates of molecular testing increased from 62% to 91%, and the availability of molecular testing results at the initial medical oncology consultation increased from 6% to 37%. However, the article does not mention the methods and statistical analyses or the sample size [46].

Battaglia et al. advanced their work in cancer patient navigation through a randomized clinical trial assessing whether integrating legal support into an existing navigation program could further enhance care delivery [34]. While the study faced limitations—most notably low accrual and the absence of a significant effect on its primary outcome of treatment timeliness—the control arm already included standard navigation, which may have reduced the measurable impact of the added legal intervention [34]. Still, a high proportion of patients began treatment within 90 days of diagnosis: among lung cancer patients, timely treatment improved from 63% with standard navigation to 88% with the enhanced model, though the small sample size limits the strength of this finding [34]. Overall, results suggest that standard navigation alone was already effective, potentially leaving limited room for further gains through legal support [34].

In Fleisher et al.‘s study [47], there was no control group, there was a small sample size (*n* = 44) and only 6% of the sample was lung cancer and did not focus on measuring timeliness in care as an outcome [47]. However, their results showed increased knowledge in cancer, a reduction in worry about diagnosis, higher scores on the importance of adhering to treatment plans, and improvements in the management of distress, financial issues, and appointments [47]. No effect was found in patient satisfaction [47].

#### Clinical outcomes

Cykert et al. [41] showed increased odds of treatment completion (1.6 OR *p* < 0.04) and increased proportion of patients receiving treatment (80% retrospective control, 83% concurrent control and 88% intervention). However, when reporting the results, the study did not discriminate between lung and breast cancer patients [41].

Pitter et al. reported on a quasi-experimental study evaluating the impact of OnkoNetwork, a hospital-based navigation system, on overall survival in patients with newly diagnosed non-small cell lung cancer [48]. The study compared outcomes before and after implementation of the program using propensity score-weighted cohorts. They initially found a significant reduction in the hazard of death in the intervention group (Hazard Ratio: 0.63, *p* = 0.039) [48]. However, after adjusting for post-baseline factors such as Eastern Cooperative Oncology Group (ECOG) performance status and disease stage at treatment initiation, the survival benefit was attenuated—suggesting that navigation may have acted indirectly by facilitating earlier diagnosis and better treatment readiness [48].

## Discussion

This lung cancer scoping review on PNP was ideal to map available evidence, examine how research is conducted and identify key characteristics related to the interventions [26, 49]. Despite the potential selection bias, small sample, and lack of focus on the minimisation of prolonged care intervals, these studies share promising evidence and could potentially lead to better results if outcomes were measured in a larger sample and more rigorous outcome evaluation.

The emergence of patient navigation as a field suggests patient navigators play an increasingly crucial role in bridging the gap between access and care. With plenty evidence over the administrative and clinical benefits of patient navigation [18], there also is a fundamental human need for patients to feel supported, and patient navigators are progressively assuming the role of the closest point of contact within the healthcare system. Due to the increasing interest in patient navigation research, World Health Organisation (WHO) has just recently published the technical brief on patient navigation for breast cancer [50]. Similar work needs to be done for lung cancer.

As indicated by the review, literature on cancer navigation is more abundant in high-income countries, irrespective of whether their health systems are privately or publicly funded. This prompts the question: why? Further research is needed to explore the reasons behind the lesser prevalence of PNP in low- and middle-income countries (LMICS) and whether the economic and health system structures play a role in shaping the global prevalence of such programmes. Alternatively, PNP might not be publishing their results in an academic environment, thus explaining why the literature in LMIC is less prevalent on PNP.

Our study shows that not all studies are RCT [22] and that there is frequent use of quasi-experimental design with no control group [28]. Altogether, these studies underscore the critical need to anticipate and address operational barriers early in the design of navigation interventions to ensure successful implementation [29–31].

Our scoping review, like previous literature, found PNP include other types of cancer in their studies [22] and do not stratify the results. For example, one article comprehensively described the trial but had considered stroke patients too [30]. Nonetheless, lung cancer patients were separated from recruitment to the analytical phase, making it easier to determine if the results were relevant or not. Thus, if a single cancer-type evaluation is not possible, stratification must be done to allow for further research to be conducted in PNP.

Moreover, PNP are sometimes paired with other interventions. For instance, some studies have paired their intervention with real-time warning systems, a certified nurse navigator, a patient registry, and web-based social determinants of health platforms to identify and address barriers to care and physical exercise in a person-centred delivery model [40–42]. Although this is quite pragmatic, using alternative study designs might help evaluate if the patient navigation itself is what is making the outcomes better—the sum of all of them together or just a part of it. Otherwise, the noise generated by the other interventions will shift the effect of different cancer outcomes.

According to our study, there is a varied spectrum of services or activities offered by PNP for symptomatic lung cancer patients. Three prominent activity themes were identified, each emphasising distinct aspects of patient care. The first theme centres around providing emotional support to patients, acknowledging the profound impact of emotional well-being on the overall healthcare experience. Programs adopting this theme prioritise activities such as emotional support, recognising the importance of addressing the psychological challenges that patients may face during their medical journey. Navigators in these programs play a crucial role in offering empathy, counselling, and support to enhance the holistic well-being of patients. The second theme encompasses a broad spectrum of activities, extending beyond emotional support to include Transportation, Legal Support, and Lobbying/Advocacy. This comprehensive approach, acknowledging that patient needs extend beyond the clinical setting. Navigators in these programs engage in diverse activities to tackle logistical, legal, and systemic challenges, advocating for policy changes and ensuring patients have access to a range of supportive services. The third theme revolves around guiding patients through the complexities of healthcare systems. By prioritising activities such as Referrals and Infrastructure Navigation, navigators in these programs serve as guides, helping patients navigate intricate healthcare processes, connect with appropriate medical services, and overcome barriers to access. This theme underscores the importance of ensuring patients receive timely and well-coordinated care within the complexities of the healthcare landscape. Collectively, these themes showcase the diversity of patient navigation approaches, although they’re designed to address specific facets of the patient experience or healthcare challenges, the inconsistency limits generalizability of each scoped work. Therefore, even if patient navigation is a tailored intervention, frameworks should aim to help PNP to measure and standardise their results.

In consequence, this scoping review points to PNP, for symptomatic lung cancer patients, still being defined. It currently represents different programmes intervening in care at different moments in the disease continuum, making it too difficult to evaluate the effects of patient navigation across the lung cancer continuum. Systematic reviews now may not be the most reliable and effective means to capture the success or failure of interventions, especially when the interventions differ significantly. Guidelines like the ones developed by WHO to support the implementation and research of patient navigation should be developed to support future evidence-based decisions through systematic reviews and meta-analyses.

Previous efforts have tried to map the outcome measures in patient navigation [51]. Results from this review suggest a consistent emphasis on administrative outcomes, potentially reflecting the prioritisation of program efficiency. The diversity found in utilising clinical and patient-reported outcomes may indicate a need for standardised approaches in these domains. Hence, this study underscores the importance of establishing a consensus on outcome selection and ensuring its dissemination, to allow for comprehensive and comparable assessments across patient navigation initiatives, fostering evidence-based practices and enhancing the overall impact of these programmes on patient care and health outcomes [51]. Furthermore, from these results, we can conclude the research agenda for early lung cancer diagnosis [52] and patient navigation [1] are not always linked by the objective of healthcare timeliness. Thus, the expansion of a shared agenda among both academic and non-academic stakeholders will further the impact PNP has in increasing diagnostic and treatment timeliness in cancer care.

In the results found, there is a pattern of excluding specific demographics. Exemplifying this trend is the conspicuous absence of individuals with a cancer history, recent cancer treatment recipients (within the last five years), those concurrently undergoing cancer treatment, individuals whose primary language is non-English, patients lacking decisional capacity, and those institutionalised, incarcerated, or afflicted with cognitive impairment (e.g., dementia or conditions induced by metabolic, medication, or drug-related factors). This underscores a methodological concern in public health research, revealing a predilection towards investigating populations perceived as optimally poised to benefit from patient navigation initiatives [53]. Consequently, the discernible proclivity to include only idealised subjects raises pertinent questions regarding research findings’ external validity and translational potential [54].

The non-reporting of comorbidity within individual studies raises substantial concerns regarding the potential repercussions on result variability across the literature. Patients with multiple comorbidities may exhibit distinct responses to navigation interventions. For instance, individuals with depression may manifest heightened disengagement during the navigation process, thereby introducing a nuanced layer to the assessment of program success [55]. The bio-psycho-social ramifications of concurrent diseases can significantly influence clinical outcomes [56]. Consequently, a comprehensive evaluation of PNP’s effectiveness necessitates meticulously considering the diverse comorbidity prevalent among the patient population under scrutiny. Failure to account for these multifaceted health dynamics could compromise the validity and generalisability of findings within the broader healthcare landscape [54].

A notable observation is that most studies fail to contribute their findings to platforms like Cochrane and the International Clinical Trials Registry Platform by WHO. This practice poses challenges for researchers engaged in systematic reviews or individuals seeking information on the topic, as locating dispersed publications becomes a cumbersome task. Some studies had multiple articles [41, 42, 57, 58], sometimes published by different authors in different years. Hence, it is important to keep the trial registry number or the title of the patient navigation programme easily reachable for the researchers. Also, it would also help if the previous published literature is outlined in the subsequent articles to make it easier to put the story together. Scoping reviews like this one, allows you to go back and forward between documents and capture the full idea of the navigation programme, its implementation, and results. Hence, it is advisable for researchers to actively return to clinical trial registry websites to ensure the posting of their results, enhancing accessibility and facilitating comprehensive reviews of the literature.

It is important to understand the nature and networks of institutions or organisations from which PNP originates. This practice could highlight potential collaboration models, including private or public-private partnerships. Such insights are increasingly crucial in the implementation of PNP. However, only one study provided a detailed account of the actors involved in the navigation programme [59]. Although actors are mentioned, their roles and contributions are unclear in the research papers. Thus, the provision of such information is encouraged.

The reliance on specific code names could impact the comprehensiveness of the literature coverage, potentially omitting relevant contributions that used alternative terminology (i.e., case navigation, case management). Despite these limitations, the chosen approach was deemed necessary to balance the need for timely updates with the practical constraints of sifting through an extensive body of literature. Researchers should exercise caution in generalising the findings beyond the defined scope and consider potential omissions resulting from the search strategy employed.

## Conclusion

This review yields specific recommendations for future research in lung cancer patient navigation for symptomatic patients. These include the imperative to homogenise implementation and research methodology, whilst also standardising outcome measurements to be able to conduct systematic reviews in the future. Specifying time intervals of interventions and stratifying results by cancer type will contribute to current or future evaluation of PNP. Other recommendations include updating clinical registry portals, enhanced transparency regarding the nature of the collaborative effort and networks, applied methodology, incorporate racial or ethnic minorities in trials to ensure comprehensive outreach to the most marginalized demographics, and elucidate the comorbidities of recruited patients. Additionally, it is important to embed a research agenda addressing health inequalities, realigning with the original purpose of patient navigation.

## Supplementary Information

Supplementary Material 1.

## Data Availability

All collected data is available in the supplementary file.

## Abbreviations

CaNDI: Cancer Needs Distress Inventory
CT: Computed tomography
EQ-5D-5L: Quality of life survey by EUROQOL
EORTC QLQ-C30: European organisation for research and treatment of cancer quality of life questionnaire
EORTC QLQ-LC13: European organisation for research and treatment of cancer quality of life questionnaire for lung cancer
LMICS: Low- and middle-income countries
PACIC: Patient assessment of chronic illness care
PET: Positron emission tomography
PRISMA- ScR: Preferred reporting items for systematic review and meta-analysis
PNP: Patient navigation programme
RCT: Randomised controlled trials
USA: United States of America
WHO: World Health Organisation
